# Can Serum Salusin-α and Salusin-β Serve as Biomarkers for Early Atherosclerotic Lesions in Obese Children?

**DOI:** 10.3390/ijms26083549

**Published:** 2025-04-10

**Authors:** Anna Medyńska, Joanna Chrzanowska, Monika Seifert, Danuta Zwolińska

**Affiliations:** 1 Department and Clinic of Pediatric Nephrology, Medical University of Wrocław, 50-367 Wrocław, Poland; danuta.zwolinska@umw.edu.pl; 2 Department and Clinic of Pediatrics, Endocrinology, Diabetology and Metabolic Diseases, Medical University of Wrocław, 50-367 Wrocław, Poland; joanna.chrzanowska@umw.edu.pl (J.C.); monika.seifert@umw.edu.pl (M.S.)

**Keywords:** salusin α and β, obesity, children

## Abstract

Obesity increases the risk of atherosclerosis. Recent studies highlight the role of salusins, bioactive peptides, in its development. This study aimed to determine the risk factors for premature atherosclerosis in obese children based on obesity severity and assess the usefulness of serum salusin-α and salusin-β as early biomarkers. We examined 125 children with simple obesity, divided into two subgroups by BMI-SDS (I: 2–4, II: >4), and we compared them with 33 healthy-weight peers. Obese children had significantly higher serum salusin-α and salusin-β levels, as well as hsCRP, TG, SBP, DBP, and PWV, compared to controls. Only salusin-α levels increased with obesity severity. Salusin-α correlated with BMI SDS and hsCRP, while salusin-β showed no associations. Our findings suggest that salusin-α could be an early inflammation marker and a predictor of atherosclerosis, pointing to salusin-α, rather than salusin-β, as the earliest marker of atherosclerosis in obese children. Further research is needed to confirm these results.

## 1. Introduction

According to the WHO European Regional Obesity 2022 report, the prevalence of obesity and overweight has increased in recent years, and it was exacerbated during the COVID-19 pandemic [1]. The problem affects almost 60% of adults and nearly 30% of children (29% of boys and 27% of girls) [1]. In Poland, the scale of this phenomenon is also alarming and continues to grow [2]. Childhood obesity can have consequences in adulthood, even if it does not persist over time. It has been shown to be a risk factor for damage to almost every system and organ, including the presentation of serious health risks such as diabetes, high blood pressure, and atherosclerosis. These, in turn, lead to cardiovascular complications and a higher risk of mortality [3]. Adipose tissue functions as a biologically active organ, producing adipokines that play a crucial role in various physiological processes. These include glucose and lipid metabolism, immune response regulation, blood pressure control, and inflammatory processes. Atherosclerosis is a chronic condition characterized by vascular endothelial damage, cholesterol accumulation in the intimal membrane, the formation of atherosclerotic plaques, and ongoing inflammation, all of which are key factors in its pathogenesis [4,5]. These changes lead to vascular stiffness and the narrowing of the vessel lumen, potentially resulting in complete occlusion. Early diagnosis of atherosclerosis using biomarkers is therefore crucial. The pathogenesis of atherosclerosis is complex, with strong evidence linking high-sensitivity CRP (hsCRP) to its development [6,7,8]. This protein inhibits nitric oxide production in the endothelium, contributing to vascular damage [9]. It also plays a role in cholesterol uptake by macrophages and their transformation into foam cells, a key factor in atherosclerosis progression [6,7].

An association between elevated hsCRP levels and cardiovascular events, as well as mortality, has been demonstrated in adult patients [10]. In obese adults with insulin resistance, increased hsCRP levels have been observed. In recent years, significant attention has been given to salusins, endogenous biopeptides with opposing effects. Salusin-α and salusin-β, consisting of 28 and 20 amino acids, respectively, are derived from preprosalusin, which is expressed in various tissues and organs, including the endothelium, muscles, nervous system, bone marrow, kidneys, plasma, and urine. The impact of salusins on atherosclerosis is primarily linked to their influence on acetyl-CoA acetyltransferase 1 (ACAT-1) protein expression, which promotes cholesteryl ester accumulation and foam cell formation [11].

The antiatherogenic effect of salusin-α is mainly related to the inhibition of foam cell formation and a reduction in the inflammatory response [12,13]. In experimental studies, Esfahani et al. showed that salusin-α decreased gene expression for pro-inflammatory cytokines such as IL-6, IL-8, and IL-18 [12] and increased the mRNA expression of the anti-inflammatory cytokine IL-1Ra in human umbilical vein endothelial cells (HUVECs), thereby suppressing pro-inflammatory IL-1 [12,13]. Additionally, salusin-α administration in animal models has been shown to reduce both atherosclerotic plaque size and hepatic steatosis [14,15]. Clinical trials have further indicated lower serum salusin-α levels in patients with coronary artery disease [16] and hypertension [17,18,19].

Conversely, salusin-β plays a proatherogenic role and is considered a more reliable indicator of atherosclerosis progression than salusin-α [20]. It exhibits both pro-inflammatory and pro-oxidant activity, promoting the release of oxygen free radicals and inflammatory cytokines such as interleukin IL-1β, IL-6, and tumor necrosis factor alpha (TNF-α). Additionally, it stimulates the proliferation of vascular smooth muscle cells, contributing to intimal hyperplasia, vascular fibrosis, and atherosclerotic lesion formation. By influencing the autonomic nervous system, salusin-β alters vascular hemodynamics, ultimately leading to hypertension [21,22]. In vivo studies on HUVECs have demonstrated that hyperglycemia increases salusin-β synthesis and causes vascular endothelial damage, a finding supported by clinical research in diabetic patients [23].

The study aimed to evaluate the risk factors contributing to premature atherosclerosis in obese children based on the severity of obesity and to assess the potential of serum salusin levels as biomarkers for early atherosclerotic lesions in this patient group.

## 2. Results

Patients in the obese group and healthy controls did not differ in age or gender. BMI, SDS-BMI, SBP, and DBP were higher in the obese group than in the controls. Detailed data are presented in Table 1.

Mean triglyceride levels were significantly higher, while HDL cholesterol levels were lower in obese children compared to their lean peers. Total and LDL cholesterol levels were slightly higher but did not differ significantly between the groups.

Mean fasting glucose levels were significantly higher in obese children. However, only four in the study group had above-normal levels, and the same patients showed abnormal results after 120 min of the glucose load test. Detailed data are presented in Table 2.

The group of obese children exhibited significantly higher levels of both types of salusin compared to their lean peers. Additionally, hsCRP levels were elevated in the obese group relative to the controls. Similarly, SDS PWV values normalized for height were higher in obese children than in their healthy counterparts. Detailed data are presented in Table 3.

**Figure 1:**
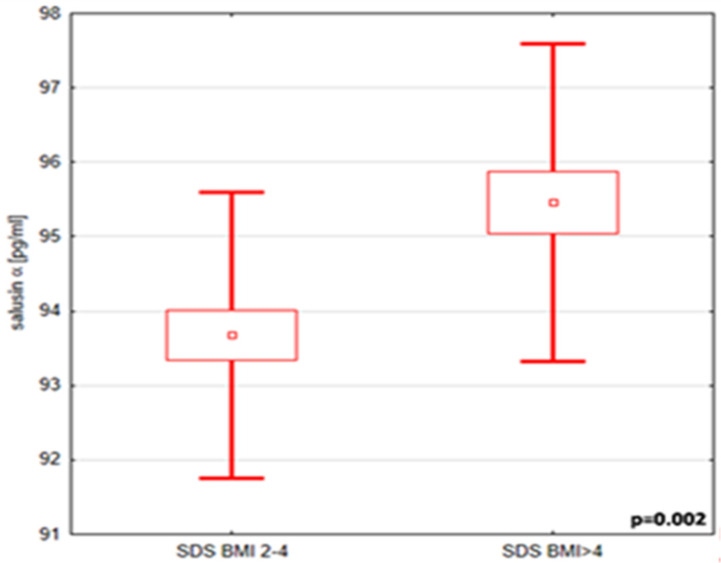
Comparison of the average salusin-α concentration in the group of children with BMI-SDS between 2 and 4 and >4 (*p* = 0.002).

Analysis based on obesity severity revealed that, aside from body weight, these subgroups differed significantly in mean diastolic pressure and salusin-α levels, both of which were higher in children with BMI-SDS > 4. Surprisingly, no significant differences were observed in lipid and carbohydrate parameters. Detailed data are provided in Table 4 and Table 5, as well as Figure 1.

**Figure 2:**
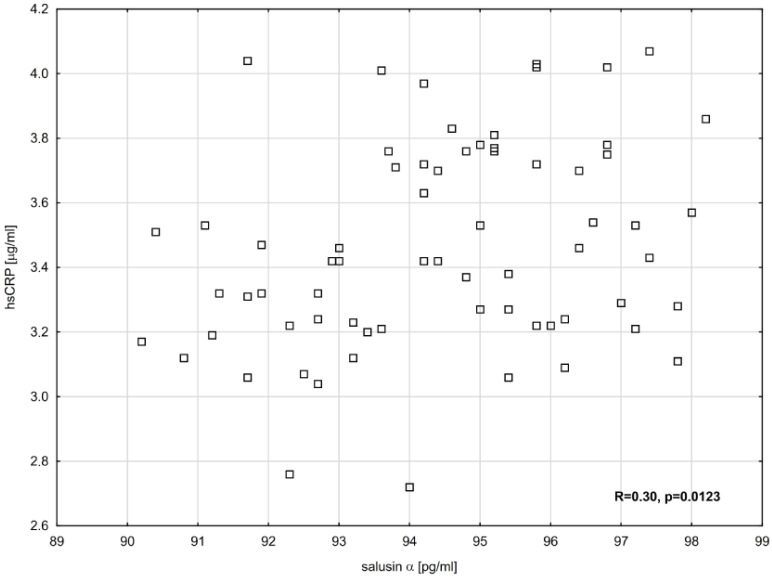
hsCRP correlation depending on salusin-α (Spearman’s correlation coefficient, R = 0.30, *p* = 0.0123).

For the entire group of obese children, significant positive correlations were found in the association between salusin-α and BMI-SDS (R = 0.32, *p* = 0.0101) and between hsCRP concentration and salusin-α (R = 0.3, *p* = 0.0123), as illustrated in Figure 2.

## 3. Discussion

There is no doubt that obesity in children and adolescents carries a high risk of developing atherosclerosis, which typically remains asymptomatic at this stage of life, making it difficult to establish clear diagnostic criteria. Assessing the known risk factors for atherosclerosis as early as possible is therefore crucial. These include conditions linked to metabolic syndrome, such as dyslipidemia, impaired glucose metabolism, hypertension, and inflammation. Recently identified factors, salusin-α and salusin-β, have been documented to play anti- and proatherogenic roles in adults. However, their significance as predictors of atherogenic indices in obese children remains unknown.

Our study found that serum salusin-β levels were significantly higher in obese children than in their normal-weight peers but showed no variation between subgroups based on SDS-BMI. Additionally, no correlation was observed between salusin-β and SDS-BMI, consistent with the findings of Dervisgulu et al. [19]. In their study of 75 obese children aged 6–18 years, they also found no association with hypertension, left ventricular mass index (LVMI), carotid intima-media thickness (CIMT), or other risk indicators for atherosclerosis and cardiovascular events [19]. However, unlike our findings, their study population did not differ from the normal-weight reference group in terms of salusin levels. Moreover, SDS-BMI levels in their study were significantly lower (2.01 ± 0.27 vs. 4.02 ± 1.7), which may explain these differences.

The association between obesity and elevated salusin-β levels was not demonstrated by Stefanowicz-Bielska et al. in a small group of patients with Down’s syndrome who, apart from higher HDL-C and uric acid levels, did not differ from normal-weight individuals [24]. Conversely, Pahoo et al. investigated the effects of 12 weeks of moderate- and high-intensity interval training (HIT) in boys over 11 years old. They found that salusin-β levels increased while salusin-α levels decreased, with both changes correlating positively with a reduction in BMI. HIT proved to be more effective in this regard. Additionally, both training regimens had a beneficial impact on the atherogenic lipid profile and inflammatory markers, as reported in two studies by Pahoo [25,26]. Similar effects related to exercise were also observed in obese women [27].

These findings suggest that salusin-β can elevate blood pressure by upregulating sympathetic nerve activity, inflammatory factors, and superoxide anion levels. Kolakowska et al. examined children with primary hypertension, of whom 37/58 (63.8%) were overweight or obese, and found higher serum salusin-β concentrations compared to normotensive children. Unlike our study, they not only established an association between BMI z-score, DBP, and SBP across three independent measurements but also identified a positive correlation between salusin-β and triglycerides, the TG/HDL-C ratio, hsCRP, and ADMA—key markers of atherosclerosis risk [28,29].

With one exception, there are no reports on salusin-α in obese adults and children. In the study by Dervisoglu et al., no difference was observed between obese and normal-weight control groups. Apart from a negative correlation with DBP, no association with other cardiovascular risk factors was found [19]. These findings contrast with ours. Our findings demonstrate higher serum salusin-α concentrations in the obese group, particularly in individuals with BMI-SDS > 4 compared to those with a lower BMI-SDS. Additionally, we found a positive association between salusin-α and BMI-SDS, but not with blood pressure.

Differences between studies may be due to the diversity of the studied populations in terms of BMI, DBP, and SBP. Our patients had higher BMI-SDS values and lower DBP and SBP levels. In adults with primary hypertension, Alpsoy et al. reported decreased serum salusin-α levels, particularly in non-dippers, along with increased salusin-β. This suggests that salusins may mediate crosstalk between the sympathetic and parasympathetic systems and serve as indicators of poor cardiovascular prognosis in hypertensive patients [30].

For the first time, we not only demonstrated elevated serum salusin-α levels in obese children but also identified its association with hsCRP (positive correlation). hsCRP is a well-established atherogenic factor and a marker of cardiovascular events, extensively documented in adults with advanced atherosclerosis, particularly in patients with coronary artery disease, where it strongly correlates with the severity of vascular changes [31]. Similarly to the findings of Roh et al., our study confirmed elevated serum levels of this protein in obese children [32]. This is likely linked to the production of pro-inflammatory cytokines by adipose tissue, especially IL-8 and TNF-α, which promote vascular inflammation and may contribute to atherosclerotic plaque destabilization. Roh et al. also reported a positive correlation between hsCRP and carotid IMT, as well as a negative correlation with brachial flow-mediated dilatation (FMD), both indicative of vascular changes [32].

In our study, we used the height-normalized PWV method for vascular assessment. Although obese children had significantly higher SDS-PWV values compared to their normal-weight peers, no correlation was found with the tested variables.

The observed positive correlation between salusin-α and hsCRP remains unclear. However, it may represent an adaptive response to increased pro-inflammatory cytokines, aiming to counteract inflammation and the proatherogenic effects of salusin-β. TNF-α, in particular, could play a key role, as it has been shown to influence the release of salusin-β from human monocytes/macrophages [33]. It is possible that salusin-α has a similar function. The absence of elevated blood pressure in our study population and the absence of an association between salusins and metabolic atherogenic indices suggest that salusin-α may help prevent further vascular and atherogenic progression. Over time, however, persistent exposure to adverse factors may disrupt this balance, leading to a decline in salusin-α and the deterioration of its protective effect. Thus, the increase in salusin-α associated with inflammation may be the first sign of ongoing changes associated with the development of atherosclerosis in obese asymptomatic children.

A limitation of this study is its small sample size, which prevented the assessment of serum salusin levels in more severe manifestations of metabolic syndrome.

## 4. Materials and Methods

This prospective cohort study included 125 children (68 girls and 57 boys) aged 8–18 years, with simple obesity, free from chronic co-morbidities and acute infectious diseases at the time of the study. Participants were recruited from the Clinic of Pediatrics, Endocrinology, Diabetology, and Metabolic Diseases at the Medical University of Wrocław, Poland. Children with secondary obesity or acute infections or those taking immunomodulatory medications were excluded.

To achieve the aims of the study, the participants were divided into two subgroups based on their body mass index standard deviation score (BMI-SDS), as follows: subgroup I comprised 65 children (BMI-SDS > 2 < 4) aged 8–17.9 years, while subgroup II included 60 children (BMI-SDS > 4) aged 8.2–17.8 years. A control group of 33 healthy peers with normal body weight and similar age was also included.

Informed consent was obtained from the parents of all participants, as well as from children over 16 years of age. The study protocol was approved by the Bioethics Committee of the Medical University of Wrocław (No. 376/2016) and conducted in accordance with the Declaration of Helsinki.

All children underwent a medical history review and physical examination. Standing height was measured to the nearest 0.1 cm using a Harpendenstadiometer, while body weight was recorded to the nearest 0.05 kg with an electronic scale (SECA, Hamburg, Germany). BMI was calculated as weight (kg) divided by height (m^2^) and expressed as a standard deviation score (SDS) for age and sex based on Polish percentile charts [34]. According to the WHO guidelines, children with a BMI exceeding 2 SDS were classified as obese.

Blood pressure (BP) was assessed using the oscillometric method with an Omron 705 IT device (Omron Healthcare Co., Kyoto, Japan) and an appropriately sized cuff under standard conditions. In accordance with the current European recommendations [35], hypertension was diagnosed in patients up to 15 years of age if systolic and/or diastolic BP values met or exceeded the 95th percentile for sex, age, and height according to Polish child population percentile charts (OLAF study calculator). For individuals aged 16 and older, hypertension was defined as BP exceeding 140/90 mmHg in at least three independent measurements.

After overnight fasting, venous blood samples were collected from each child and centrifuged at 3000 rpm for 15 min. The separated serum was then frozen at −80 °C and stored until analysis. Serum levels of total cholesterol, HDL cholesterol, LDL cholesterol, triglycerides, and fasting glucose were measured in all patients. In obese children, insulin levels were assessed, an oral glucose tolerance test was performed, and HOMA-IR was calculated as an indirect marker of insulin resistance. Additionally, serum concentrations of salusin-α, salusin-β, and high-sensitivity CRP were determined.

Serum hsCRP was quantified using a commercially available ELISA kit (Demeditec Diagnostics GmbH, Kiel, Germany; catalog number DE740011), with a method sensitivity of 0.02 μg/mL. Salusin-α levels were measured with an ELISA kit from Wuhan Fine Biotech Co. (East Lake High-tech Development Zone, Wuhan, China; catalog number EH3741), with a sensitivity of <46.875 pg/mL. Salusin-β concentrations were assessed using an ELISA kit from Cloud-Clone Corp (Katy, TX, USA; catalog number CEC026Hu), with a sensitivity of 1.75 pg/mL.

### 4.1. Pulse Wave Velocity Measurement

Arterial blood pressure was first measured in the children. Then, with the child in a supine position, PWV recording points were marked over the carotid and femoral arteries. The straight-line distance of these points from the sternal notch was measured. Pulse wave velocity was assessed using a tonometric transducer (Miller Instruments, Inc., Houston, TX, USA) connected to a SphygmoCor recorder (AtCor Medical Pty. Ltd., Sydney, Australia) and a computer equipped with the corresponding signal analysis software.

By placing the tonometer head directly on the skin over the artery, a signal was obtained. Once a high-quality recording was confirmed by the software, pulse wave data were collected for approximately 30 s. The first measurement, taken over the internal carotid artery, was considered proximal to the heart, while the second, taken over the femoral artery, was considered distal. The test was conducted three times, and the mean value was calculated.

Pulse wave velocity was determined using the formula PWV = ds/dt, where ds represents the difference in distance between the femoral and carotid measurement points from the sternal notch (in meters), and dt denotes the time difference between consecutive R-waves in the ECG recording and the bases of corresponding pulse waves (in seconds). Due to the influence of height on pulse wave measurements, SDS-PWV was calculated. All measurements were performed in person.

### 4.2. Statistical Analysis

The mean (x), median (M), range (min–max), lower and upper quartiles (25Q–75Q), and standard deviation (SD) of the studied continuous parameters were calculated for all groups. The Mann–Whitney U test was used to assess the differences between independent groups.

Regression analysis was conducted for selected parameter pairs by calculating Spearman’s correlation coefficient. A significance level of *p* ≤ 0.05 was considered statistically significant. Statistical analysis was performed using the EPIINFO software package, version 7.1.1.14 (released on 7 February 2013).

## 5. Summary

We found significantly higher serum concentrations of salusin-α and salusin-β, along with elevated TG, SDS PVW, SBP, and DBP levels in obese children compared to their normal-weight peers. However, BP values remained within normal limits. The positive correlation between salusin-α and both BMI-SDS and hsCRP, without associations with other proatherogenic indices, suggests that probablysalusin-α may serve as an early response to inflammation. This indicates that salusin-α, rather than salusin-β, could be the earliest marker of atherosclerosis development in children with simple obesity. Further studies in a larger population of obese children are needed to confirm this hypothesis.

## Figures and Tables

**Table 1:** Characteristics of the study and control groups.

Variable		Control Group F/M 18/15	Study Group F/M 68/57	*p*
Age [years]	mean ± SD range (min–max)	12.9 ± 3.0 7.6–17.8	13.7 ± 2.84 8.0–17.9	0.172
Body weight [kg]	range (min–max) median quartile (25–75Q)	47.7 ± 11.9 48.1 38.6–55.4	85.7 ± 23.8 82.7 72.7–100	0.0001 *
BMI	range (min–max) median quartile (25–75Q)	19.2 ± 2.3 19 17.7–20.3	32.1 ± 5.8 30.8 28.4–35.2	0.0001 *
BMI-SDS	range (min–max) median quartile (25–75Q)	0.061 ± 0.633 0.061 (−0.547)–0.563	4.02 ± 1.7 3.55 2.88–5.13	0.0001 *
SBP [mmHg]	mean ± SD range (min–max)	106.3 ± 8.9 85–120	117.2 ± 9.9 98–140	0.0001
DBP [mmHg]	mean ± SD range (min–max)	65.3 ± 7.2 48–76	71.8 ± 8.0 50–92	0.0001

* analysis with the non-parametric Mann–Whitney U test. SD, standard deviation. SDS, BMI standard deviation score body mass index. SBP, systolic blood pressure. DBP, diastolic blood pressure.

**Table 2:** Biochemical parameters in study and control groups.

Variable		Control Group	Study Group	*p*
Total cholesterol [mg/dL]	mean ± SD range (min–max)	164.1 ± 14.8 133–188	179.2 ± 134.9 111–1611	0.537
HDL cholesterol [mg/dL]	mean ± SD range (min–max)	59 ± 9.6 34–78	42.2 ± 8.4 27–65	0.0001
LDL cholesterol [mg/dL]	mean ± SD range (min–max)	94.1 ± 14.9 65–121	100.4 ± 24.4 49–184	0.175
Triglycerides [mg/dL]	range (min–max) median quartile (25–75Q)	57–120 94 74–105	39–469 107 83.5–141	0.0021 *
Creatinine [mg/dL]	mean ± SD range (min–max)	0.736 ± 0.144 0.54–1.19	0.629 ± 0.123 0.37–0.89	0.0001
Fasting glucose [mg/dL]	range (min–max) median quartile (25–75Q)	75–94 88 85–91	56–153 82 77–82	0.0118 *

* Analysis with the non-parametric Mann–Whitney U test. SD, standard deviation.

**Table 3:** Serum salusin-α, salusin-β, hsCRP, and SDS PWV in the study and control groups.

Variable		Control Group	Study Group	*p*
Serum salusin-α [pg/mL]	range (min–max) median quartile (25–75Q)	79.1–83.7 81 80–81.8	90.2–98.2 94.5 92.8–96.1	0.0001 *
Serum salusin-β [pg/mL]	range (min–max) median quartile (25–75Q)	78.7–90.4 81.9 80.2–84.9	136.7–163.4 144.7 140.4–152.5	0.0001 *
hsCRP [μg/mL]	range (min–max) median quartile (25–75Q)	1.02−1.45 1.2 1.14-1.32	2.36–4.46 3.22 2.86–3.54	0.0001 *
SDS PWV	range (min–max) median quartile (25–75Q)	(−4.08)–0.97 −1.31 (−2.7)–(−0.53)	(−9.43)–2.33 −0.16 (−1.0)–0.46	0.0106

* Analysis with the non-parametric Mann–Whitney U test. hsCRP, high-sensitivity C-reactive protein. SDS PWV, standard deviation score Pulse Wave Velocity.

**Table 4:** Patients’ characteristics and selected biochemical parameters according to the BMI-SDS values.

Variable		2 ≤ BMI-SDS ≤ 4 n = 65	BMI-SDS > 4 n = 60	*p*
Age [years]	mean ± SD range (min–max)	13.7 ± 2.8 8–17.9	13.5 ± 2.9 8.2–17.8	0.729
Body weight [kg]	range (min–max) median quartile (25–75Q)	34–112.2 78.2 70.5–88.4	38–170.9 99.3 79.9–113.3	0.0001 *
SBP [mmHg]	mean ± SD range (min–max)	116.1 ± 9.4 98–140	119.3 ± 10.4 99–140	0.0959
DBP [mmHg]	mean ± SD range (min–max)	70.3 ± 7.7 50–90	73.9 ± 7.9 59–92	0.0172
Total cholesterol [mg/dL]	mean ± SD range (min–max)	170.5 ± 30.9 121–259	193 ± 208.8 111–1611	0.414
HDL cholesterol [mg/dL]	mean ± SD range (min–max)	42.8 ± 8.2 27–64	40.1 ± 8.8 27–65	0.111
LDL cholesterol [mg/dL]	range (min–max) median quartile (25–75Q)	49–184 101 81–123	58–142 99 82–115	0.314 *
Triglycerides [mg/dL]	mean ± SD range (min–max)	119.2 ± 57.8 52–369	131.8 ± 70.1 39–469	0.312
Fasting glucose [mg/dL]	mean ± SD range (min–max)	82.5 ± 11.9 65–153	82.6 ± 9.1 56–105	0.952
HOMA-IR	mean ± SD range (min–max)	3.3 ± 2.87 0.03–19.76	3.89 ± 2.28 0.96–12.13	0.258

* Analysis using the non-parametric Mann–Whitney U test. SD, standard deviation. SDS, BMI standard deviation score body mass index. SBP, systolic blood pressure. DBP, diastolic blood pressure. HOMA-IR, homeostatic model assessment for insulin resistance.

**Table 5:** Serum salusin-α, salusin-β, hsCRP, and SDS PWV according to BMI-SDS values.

Variable		2 ≤ BMI-SDS ≤ 4	BMI-SDS > 4	*p*
Serum salusin-α [pg/mL]	mean ± SD range (min–max)	93.7 ± 1.9 90.2–97.8	95.5 ± 2.1 90.8–98.2	0.00146
Serum salusin-β [pg/mL]	mean ± SD range (min–max)	146.5 ± 7 137.2–162.5	146 ± 8 136.7–163.4	0.781
hsCRP [µg/mL]	mean ± SD range (min–max)	3.17 ± 0.41 2.36–4.08	3.31 ± 0.45 2.55–4.64	0.103
SDS PWV	mean ± SD range (min–max)	−0.425 ± 1.91 (−9.43)–2.33	−0.874 ± 2.117 (−7.937)–1.34	0.371

## Data Availability

Data are contained within the article.
